# Singapore’s efforts to achieve measles elimination in 2018

**DOI:** 10.5365/wpsar.2019.10.4.002

**Published:** 2021-07-12

**Authors:** Wanhan See, Yi Kai Ng, Lin Cui, Yuske Kita, Steven Peng-Lim Ooi, Vernon Lee, Derrick Mok Kwee Heng, Raymond Tzer Pin Lin

**Affiliations:** a Communicable Diseases Division, Ministry of Health, Singapore.; b National Public Health Laboratory, National Centre for Infectious Diseases, Singapore.; c Saw Swee Hock School of Public Health, National University of Singapore, Singapore.; d Infectious Disease Research and Training Office, National Centre for Infectious Diseases, Singapore.; e Public Health Group, Ministry of Health, Singapore.

## Abstract

The World Health Organization verified that Singapore had eliminated endemic transmission of measles in October 2018. This report summarizes the evidence presented to the Regional Verification Commission for Measles and Rubella Elimination, comprising information about immunization schedules; laboratory testing protocols and the surveillance system; and data on immunization coverage and the epidemiology of cases. Between 2015 and 2017, a total of 246 laboratory confirmed cases of measles were reported. The source or country of infection was unknown for most cases (195; 79.3%). There were 22 clusters, ranging from two to five cases. The most common genotypes detected were D8 and D9. Transmission of B3 was interrupted in 2017, and H1 cases were sporadic and imported. Phylogenetic analyses of the D8 isolates showed the existence of 13 lineages or clusters. Although a few lineages were circulating concurrently, no lineage propagated continuously for a prolonged period, and transmission of each lineage eventually stopped. Although cases and clusters were reported yearly, molecular data showed that none of the lineages resulted in prolonged transmission. There were fewer measles cases in 2017 compared with 2016. The higher number of clusters was likely due to the overall increase in cases because cluster sizes remained small. The occurrence of small clusters is not unexpected since measles is highly infectious. The majority of imported cases did not result in secondary transmission. With the global increase in the number of measles cases, Singapore needs to stay vigilant and continue to promptly test suspected cases; vaccination is the key to preventing infection.

The World Health Organization (WHO) reported 413 308 confirmed measles cases in 187 Member States as of 5 November 2019. (*1*) This was the fourth successive yearly increase since 2016: a total of 353 236 cases were recorded in 2018, double the number reported in 2017. (*2*) Outbreaks were occurring in countries with low immunization coverage, as well as in countries with high national vaccination rates.

The consecutive increases were a setback to the progress made towards measles elimination. The World Health Assembly endorsed the Global Vaccine Action Plan in 2012, and measles was targeted for elimination in five WHO regions by 2020. (*3*) WHO defines measles elimination as the absence of endemic transmission in a defined geographical area for more than 12 months in the presence of a well performing surveillance system. Progress towards measles elimination has varied by WHO region. In the European Region, 37 of 53 countries had eliminated measles by 2017. (*4*) Five of 11 countries in the South-East Asia Region and 9 of 37 countries in the Western Pacific Region had also achieved elimination status by 2017. (*5*, *6*) However, in the Region of the Americas, despite verification that measles had been eliminated in September 2016, the Region reported its highest increase in cases in 2017, and endemic transmission of measles had been re-established in Venezuela. (*7*) No countries in the African Region and the Eastern Mediterranean Region were verified as having eliminated measles.

WHO verified that Singapore had eliminated endemic transmission of measles in October 2018. This paper documents the immunization and surveillance systems for measles and the evidence used to achieve elimination status.

## Methods

Singapore followed WHO’s definition of measles elimination for the 2018 verification exercise. For verification purposes, countries need to provide evidence to fulfil WHO’s assessment criteria: documentation of the interruption of endemic measles and rubella virus transmission for a period of at least 36 months from the last known endemic case; the presence of verification standard surveillance; and genotyping evidence that supports the interruption of endemic transmission. (*8*) This documentation was collated by the Ministry of Health (health ministry) for the period of 2015 to 2017, in consultation with Singapore’s National Verification Committee, and included descriptions of the national immunization schedule, surveillance system and laboratory testing protocols as well as analysis of immunization coverage, the incidence and epidemiology of measles cases, and molecular analysis. These seven areas are presented in this paper.

### Ethics statement

No ethics approval was required. Data were collected under the Infectious Diseases Act of Singapore (1976), and only unidentifiable data were used for analysis.

## Results

### National immunization schedule

Measles vaccinations were first introduced in Singapore for children in October 1976, becoming legally compulsory in 1985 for children aged 1–2 years under the Infectious Diseases Act (1976). The cost of vaccination is covered by the health ministry at designated government health-care facilities. In the 1990s, when the global numbers of measles cases were high and transmission occurred even in countries with high immunization rates, many countries, including Singapore, adopted a two-dose schedule. (*9*, *10*) The timeline of changes to Singapore’s measles immunization schedule is summarized in  **Table 1**.

**Table 1:** Timeline of changes to the measles vaccination schedule in Singapore, 1976–2011

Year	Changes
1976	Introduction of the one-dose monovalent measles vaccine for children aged 12 to 24 months
1985	Measles immunization becomes compulsory for entry into primary school
1990	Monovalent measles vaccine is replaced with the trivalent MMR vaccine
1997	Catch-up vaccination programme introduced in primary schools for unvaccinated children
1998	Introduction of second dose of MMR vaccine to children in primary school at age 11 to 12 years
2008	The age for the second dose of MMR vaccine is lowered to 6 to 7 years
2011	The age for vaccination is lowered to 12 months for first dose and 15 to 18 months for second dose

MMR: measles, mumps and rubella

### Surveillance system

Measles became a notifiable disease under the Infectious Diseases Act (1976) in October 1980. In line with WHO’s guidance, the health ministry enhanced its measles surveillance in 2012 to proactively test clinical cases not swabbed by clinicians. (*11*) Medical practitioners are required to report suspected measles cases to the health ministry within 24 hours from the time of clinical suspicion, and laboratories are required to report within 24 hours of confirmation. All notifications (laboratory confirmed and clinically diagnosed) are investigated, and laboratory testing is offered to clinical cases if their symptoms fit the case definition. (*7*) The clinical case definition of measles is fever and rash with cough or coryza or conjunctivitis.

Information is collected from cases during interview and includes the date of onset of symptoms, history of exposure, travel history and vaccination records. After the investigation and laboratory testing are completed, the case is classified as a confirmed case, clinical case or not a case.

Contact tracing is initiated if confirmed cases reside in dormitories or other institutions or attend school or childcare. Public health advice is provided to all cases, and vaccination is recommended to unimmunized contacts. As per WHO’s recommendation, (*12*) the health ministry offers post-exposure chemoprophylaxis in the form of the measles, mumps, rubella vaccine or immunoglobulin to high-risk contacts at designated hospitals.

### Laboratory testing protocols

After testing specimens and if the residual samples are sufficient, public hospital laboratories are required to send an aliquot of measles (blood or oral swab or both) tested by immunoglobulin M (IgM) and polymerase chain reaction (PCR) to the National Public Health Laboratory for testing for both measles and rubella. Respiratory samples are tested concurrently for measles and rubella by PCR; if the sample is positive, this is followed by genotyping using the N-450 nucleocapsid region of the virus, as recommended by WHO. Similarly, blood samples are concurrently tested for measles and rubella by IgM. PCR testing is performed on blood samples that are IgM positive if there is sufficient serum for RNA extraction. For the cases not tested by clinicians, oral swabs are collected by the health ministry and dispatched to the National Public Health Laboratory for testing.

### Immunization coverage

The yearly immunization rate refers to national coverage of dose 1 of measles vaccine for resident children at age 2 years. In 2011, delivery of dose 2 was lowered from age 12 years to age 15 to 18 months, in accordance with changes to the schedule. These data are collected through the National Immunization Registry.

**Figure 1:**
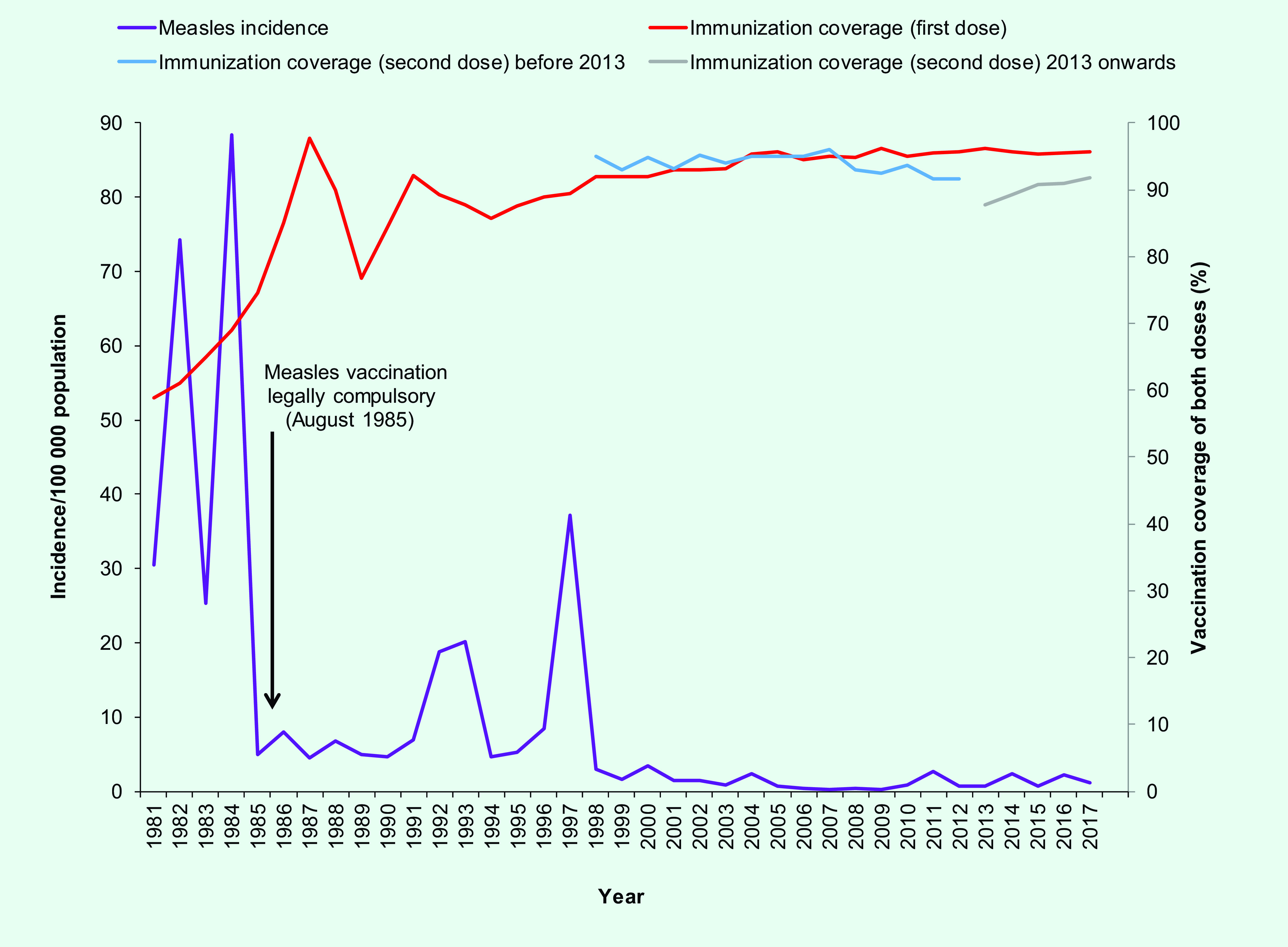
Incidence of measles and vaccination coverage rates, Singapore, 1981–2017^a^

When first introduced in the 1970s, the uptake rate of measles vaccination was low, as most people perceived that developing natural infection was an essential part of growing up. (*13*) Coverage gradually increased in the 1980s, especially after vaccination became compulsory in 1985, and it has been maintained at more than 90% since 1998 (**Fig. 1**). Coverage for the second dose has remained high since it was first rolled out. However, coverage decreased to below 90% in 2013, following a change in the schedule in 2011, which moved the first dose from being delivered at 12 to 24 months to being delivered at 12 months and the second dose from being delivered at age 6 to 7 years to being delivered at 15 to 18 months (**Table 1**). The National Immunization Registry, part of the Health Promotion Board, sends reminder letters to parents of children who miss their scheduled vaccinations.

[**insert** Figure 1]

The overall seroprevalence for measles among the resident population aged 6 months to older than 45 years was 91.4% in 1989/1990, 91.5% in 1993 and 77.9% in 1998. (*14*)

### Incidence

Measles incidence is calculated using the number of confirmed cases among local residents that were notified to the health ministry, per 100 000 population. Information on measles incidence and immunization rates includes historical data from 1981 to 2017.

Before 1985, when immunization coverage was low, measles incidence was as high as 88.5 cases/100 000. Thereafter, incidence declined as vaccination uptake improved. However, whenever there was a drop in coverage, the incidence increased two to three years later, as demonstrated in 1992, 1993 and 1997  (**Fig. 1**).

Following the introduction of the catch-up vaccination programme in 1997, measles incidence declined further to 2.9 cases/100 000 population in 1998. Since then, the incidence has remained below 4.0 cases/ 100 000 (**Fig. 1**).

### Epidemiology of measles cases, 2015 to 2017

Epidemiological information for measles cases notified to the health ministry from January 2015 to December 2017 was analysed. All notified cases were investigated, with specimens tested and classified according to the health ministry’s case definitions and WHO’s classification system. (*6*, *15*) The source of infection was either imported if links were established with a case outside of Singapore or had recent travel history, import-related if they had confirmed epidemiological links to an imported case or unknown if links were unable to be established to other confirmed cases.

**Figure 2:**
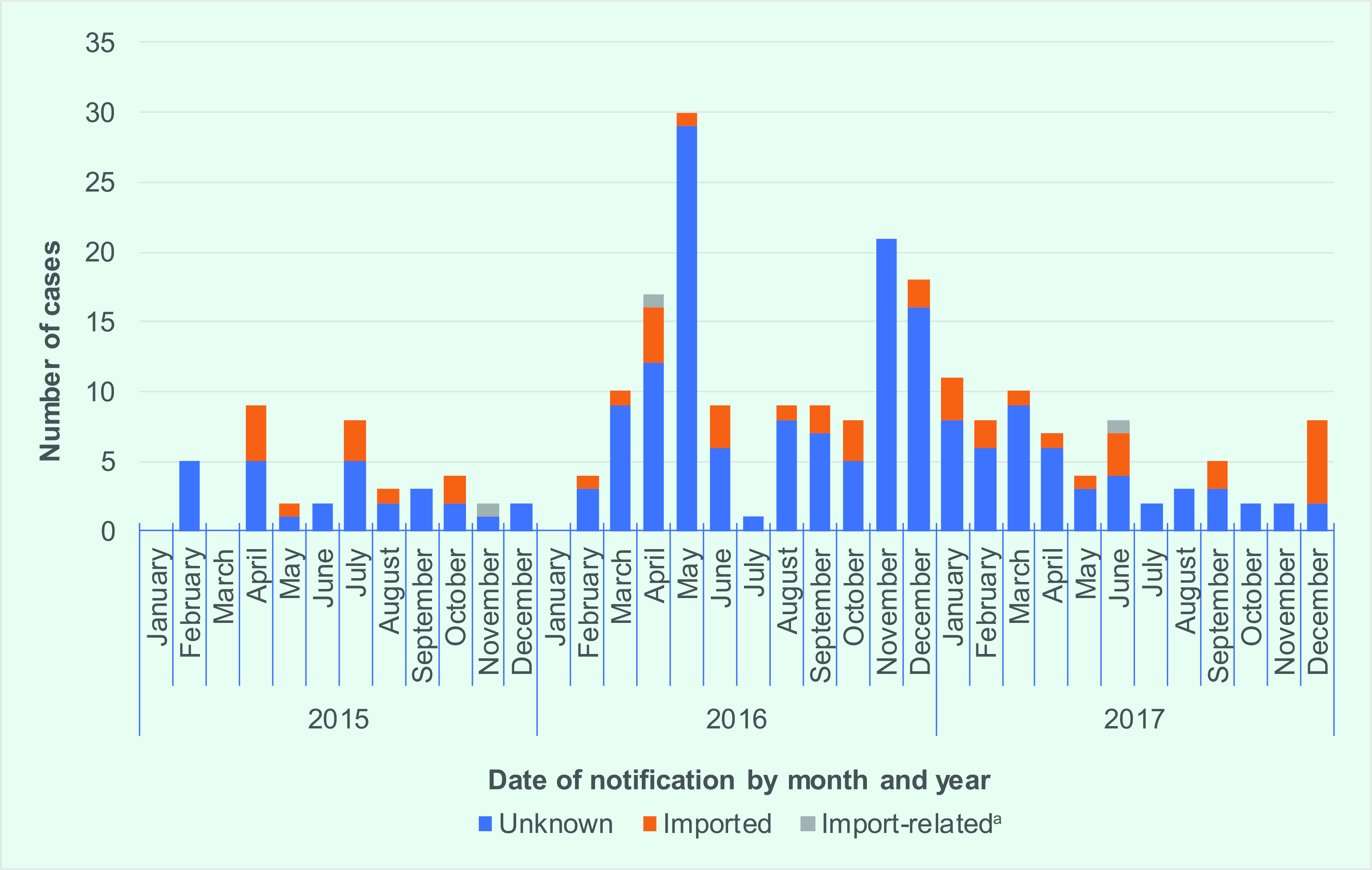
Source of infection for laboratory confirmed measles cases, Singapore, 2015–2017

Between 2015 and 2017, a total of 246 laboratory confirmed cases were reported, with the highest number of cases recorded in 2016 (**Table 2**). (*16*, *17*) During 2016, the number of cases increased from March and peaked in May, with a second peak observed in November (**Fig. 2**).

[**insert** Figure 2]

**Table 2:** Number of laboratory confirmed measles cases, clusters and genotypes reported in Singapore,  2015–2017

Number of measles cases, clusters and genotypes	Year		
	2015	2016	2017
Laboratory confirmed cases by source of infection	40	136	70
Unknown	28	117	50
Imported	11	18	19
Import-related^a^	1	1	1
Clusters	3	14	5
Samples genotyped	21	103	42
B3	1	37	0
D8	12	57	27
D9	7	8	14
H1	1	1	1

^a^ Import-related refers to a confirmed case with epidemiological links to an imported case.

**Figure 3:**
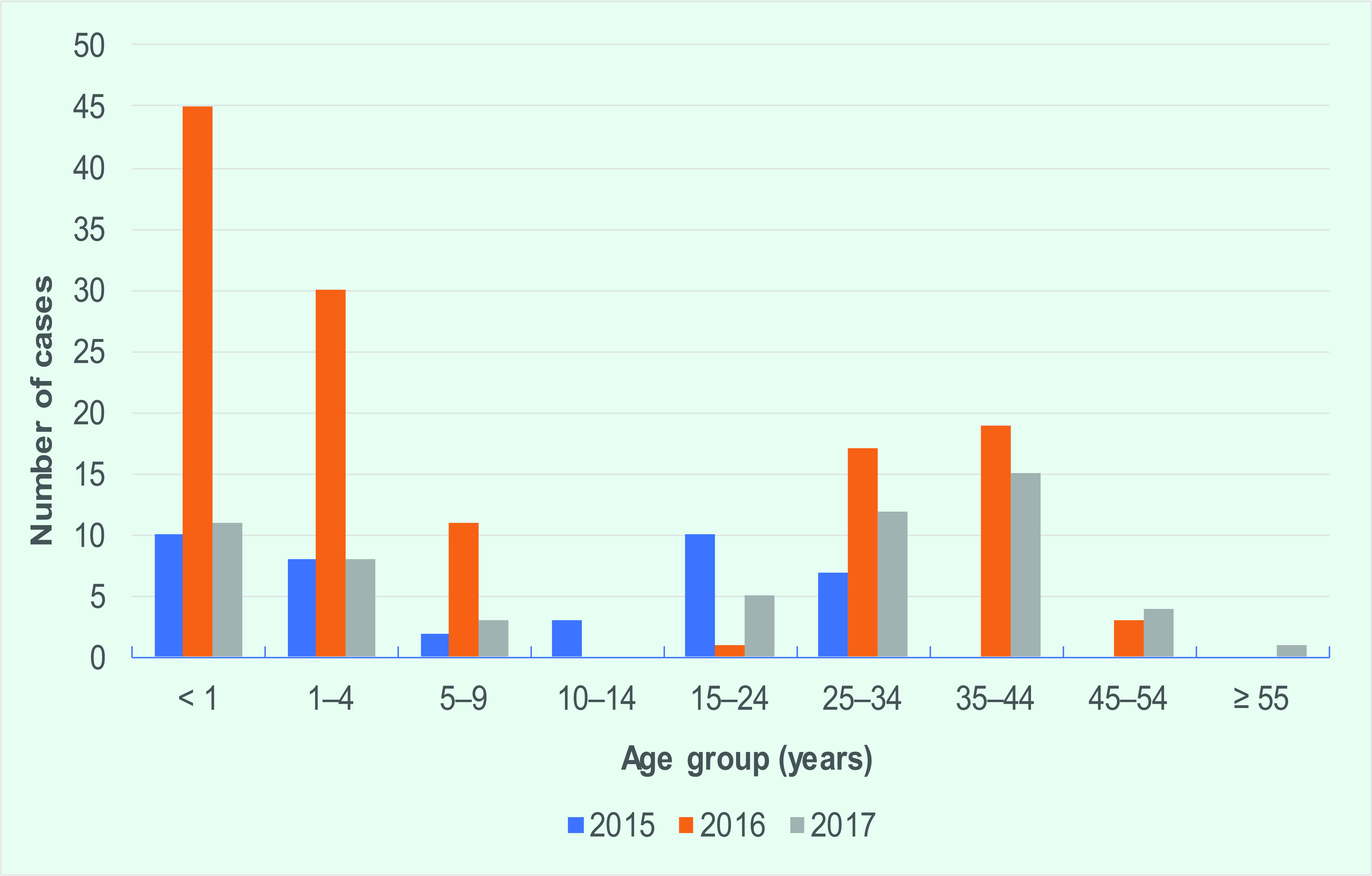
Age distribution of laboratory confirmed measles cases, by age group, Singapore, 2015–2017^a^

The age groups with the highest proportion of cases were those aged £4 years and 25–44 years  (**Fig. 3**). The male to female case ratio was approximately 1:1 for all age groups.

[**insert** Figure 3]

For most cases (195/246; 79.3%), the source or country of infection was unknown (**Table 2**). Three import-related cases were family members of a case, suggesting that most imported cases did not result in secondary transmission (**Fig. 2**). Of the 48 imported cases, the majority had travelled to or arrived from Indonesia (21; 43.8%) or Malaysia (9; 18.8%).

A total of 22 clusters were reported, and the size of each cluster ranged from two to five cases (**Table 2**). The largest cluster was reported in 2017, involving a French family of one adult and four children. All cases had similar onset dates and developed symptoms before arrival in Singapore. The family had a history of exposure to a known measles case and either had not been vaccinated or were unsure of their vaccination history. Genotyping of the respiratory samples obtained from the family were positive for D8.

### Molecular analysis

Genotypes were identified based on the N-450 sequences of measles specimens obtained from confirmed cases from 2015 to 2017. The sequences obtained from January 2016 to June 2018 were further analysed by constructing a maximum likelihood tree with 500 bootstraps using MEGA7 (Molecular Evolutionary Genetics Analysis, version 7.0). (*18*) Measles genotype D8 isolates from the same cluster or lineage were given a group number to distinguish them from other measles D8 clusters or lineages. No group number was given to D8 isolates that were phylogenetically distinct and did not form a cluster. Molecular epidemiology was used to retrospectively chart the grouped clusters based on the date of onset of rash to identify the D8 lineages circulating between January 2017 and June 2018.

**Figure 4:**
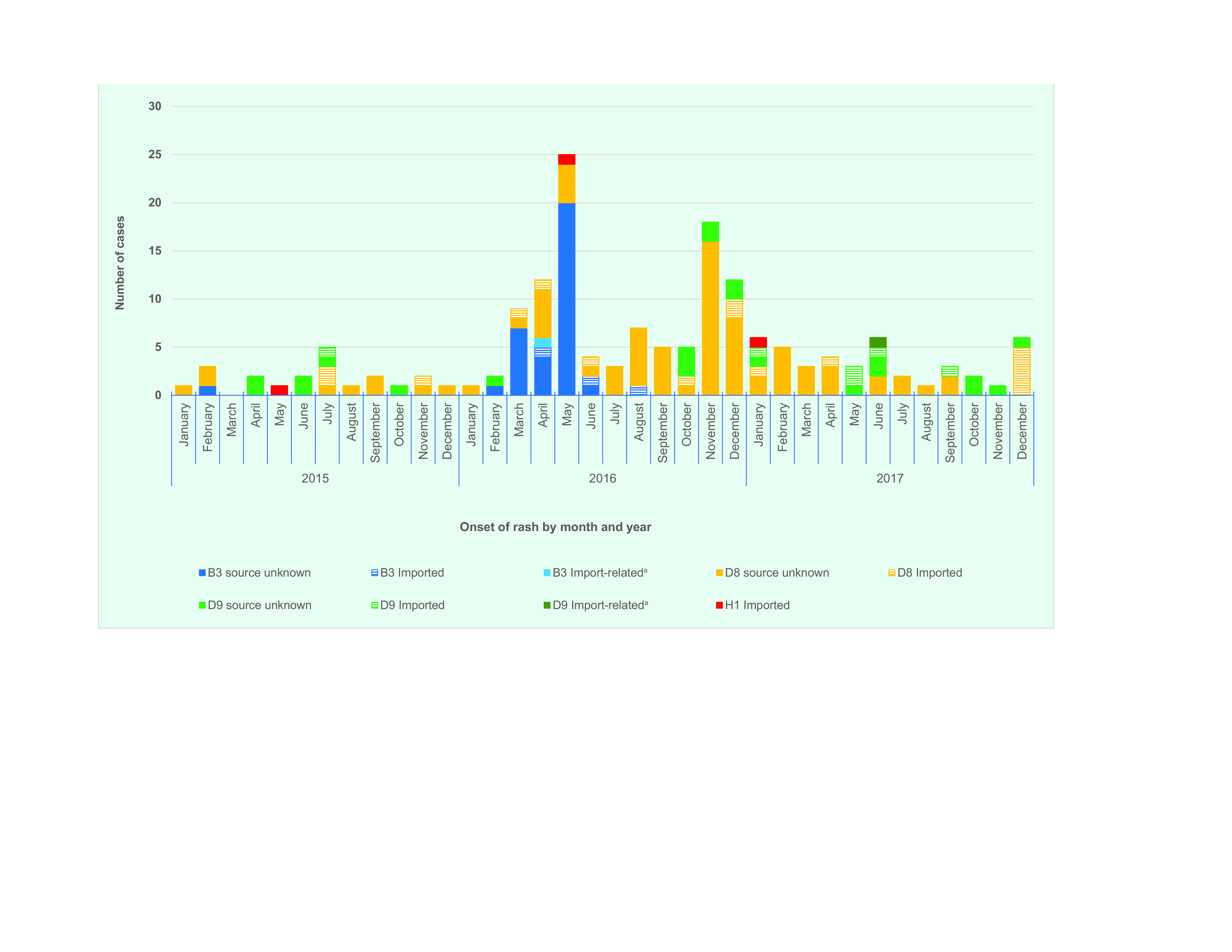
Distribution of measles genotypes by source of infection and date of onset of rash, Singapore, 2015–2017

Genotype results were available for 166 cases (67.5%) (**Table 2**), and the most commonly detected genotype was D8 (96; 57.8%), followed by D9 (29; 17.5%) (**Fig. 4**). An increase in the B3 genotype was observed between February and May 2016, with no further cases in 2017. None of the imported H1 genotype cases resulted in secondary transmission.

[**insert** Figure 4]

The maximum likelihood tree of measles genotypes is shown in **Fig. 5a**. Phylogenetic analyses of the D8 isolates showed that 13 lineages or clusters occurred from 2016 to mid-2018 (**Fig. 5b**). The D8 phylogenetic tree clusters isolates from cases that were epidemiologically linked together. Although the imported cases were found to be genetically clustered with cases from an unknown source, our investigations could not establish links between the cases. (*15*) When the onset dates were compared, some of the imported cases occurred after the cases from an unknown source; hence, it is unlikely that these cases were directly linked (**Fig. 6**).

**Figure 5a:**
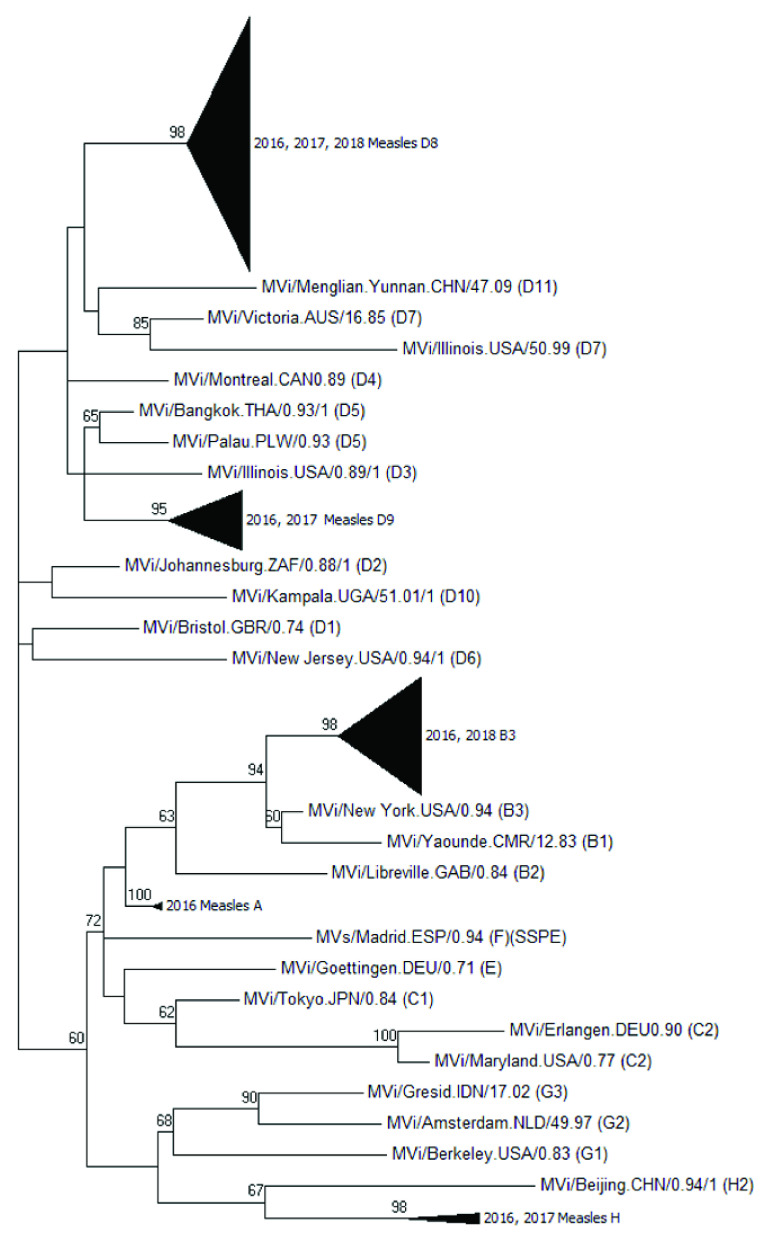
Maximum likelihood tree, measles genotypes, Singapore, 2016 to mid-2018

[**insert** Figure 5a]

**Figure 5b:**
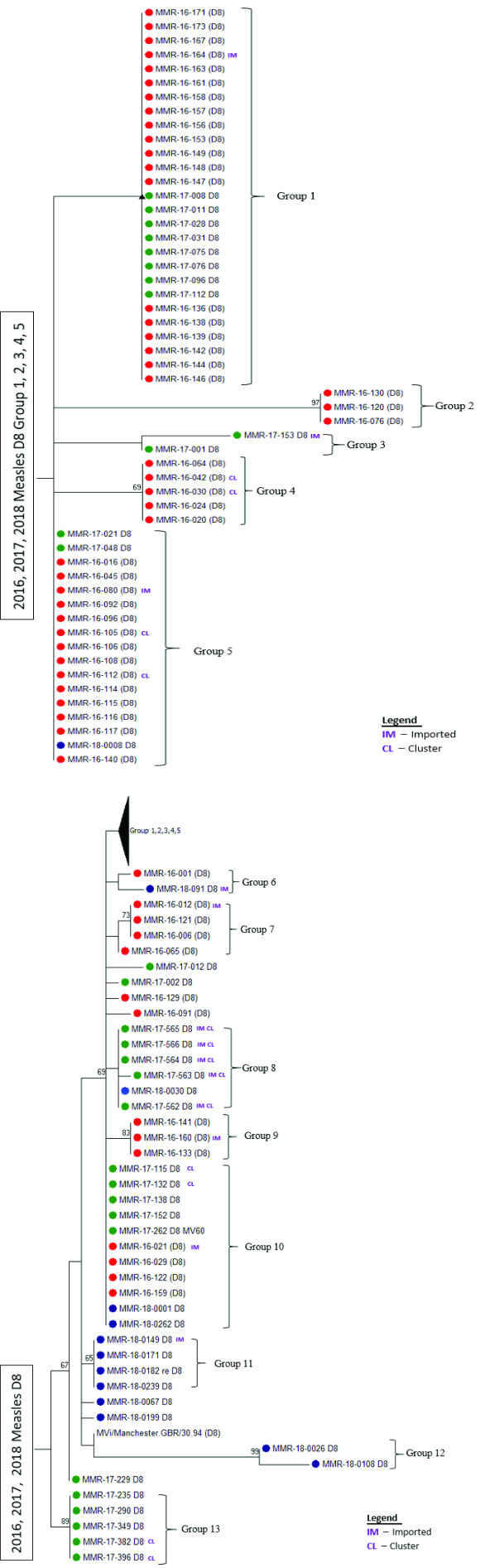
Maximum likelihood tree, measles genotype D8, Singapore, 2016 to mid-2018

[**insert** Figure 5b]

**Figure 6:**
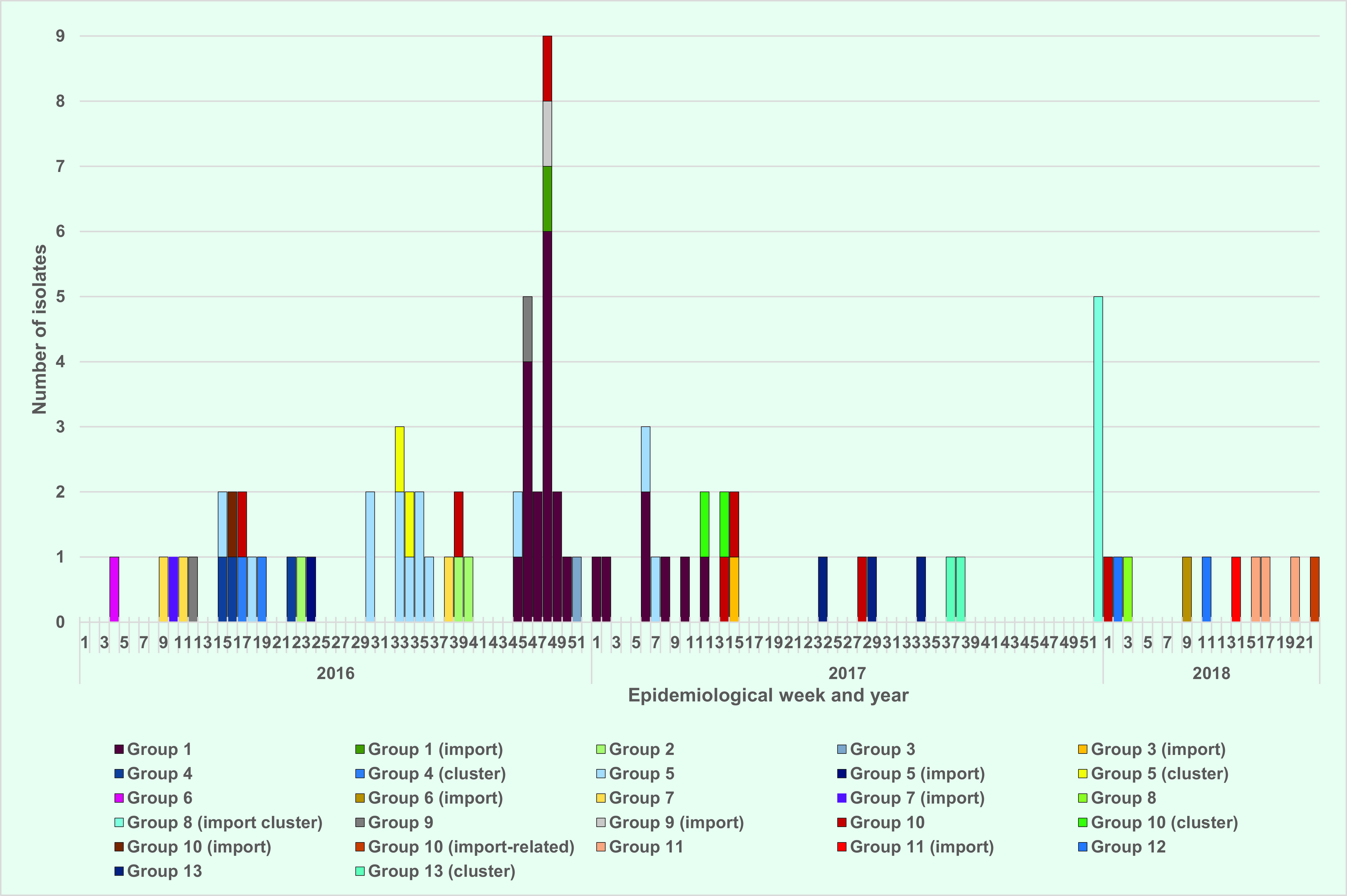
Distribution of measles D8 lineages by epidemiological week of onset of rash, Singapore, 2016 to mid-2018

[**insert** Figure 6]

An analysis of cases from the 13 D8 lineages showed that although a few lineages were circulating concurrently at one point, no lineage propagated continuously for a prolonged period (> 12 months) (**Fig. 6**).

## Discussion

Our analysis provides the evidence for Singapore to be verified as having measles elimination status – there is a well performing surveillance system for measles and an absence of endemic transmission for more than 12 months. Although measles cases and clusters were reported yearly, molecular data suggested that none of the lineages resulted in prolonged transmission within Singapore.

A variety of genotypes were detected in Singapore, similar to other countries in the Western Pacific Region. (*10*, *19*) Supported by phylogenetic analysis of D8 lineages, our data show that endemic transmission of measles did not occur in Singapore during 2015–2017. The N-450 nucleotide sequences from the nucleocapsid required by WHO’s measles nucleotide surveillance (or MeaNS) database provide adequate phylogenetic resolution to resolve the groupings or lineages for D8. However, this method does not provide the same phylogenetic resolution for the rest of the genotypes. Hence, moving forward, it is recommended to consider sequencing the extended window of the M-F non-coding region of the measles genome or perform whole genome sequencing on other common genotypes to provide molecular evidence to support case investigations and trend analyses. (*20*)

The increase in cases observed in 2016 did not continue into 2017, and the higher number of clusters in 2016 was likely due to the increase in cases as cluster sizes remained small. The occurrence of small clusters is not unexpected since measles is highly infectious. In addition, most imported cases did not result in secondary transmission.

The proportion of cases by age group did not differ greatly across the three years. The highest proportion of cases occurred among children aged £4 years. Other than those who were < 12 months of age, and thus were below the recommended age for vaccination, the remaining cases had either received only one dose (50%) or did not receive any dose (50%). The herd immunity threshold for measles is estimated at 92% to 95%. (*15*) Although from 2013 to 2017 Singapore’s immunization coverage for the first dose of vaccine remained high at 95% among children at 2 years of age, the coverage for the second dose was lower, at 86% to 90%.

A high number of cases occurred in the 25–44 year age group who were born in the 1970s and 1980s. This group could be more susceptible due to low immunization coverage in the 1970s, missing out on the second dose of vaccine or a lack of immunity among unvaccinated foreign-born individuals. However, the adult seroprevalence survey conducted on citizens in 2005 (based on residual blood samples obtained from the National Health Survey 2004) revealed that the seropositivity for this age group was higher than 95.8%. (*14*)

Because the global number of measles cases continues to increase and since Singapore is a travel hub, having received 17 million international tourists in 2017, Singapore needs to stay vigilant to detect and manage measles cases. The need for prompt testing of cases at the first suspicion should be reinforced among health-care providers to ensure that true cases are identified early. This will allow for immediate implementation of public health measures, such as isolation in hospitals or physical distancing at home. Furthermore, a higher testing rate may increase the sample size available for genotyping. Because vaccination is the key to preventing infection, efforts to advocate for immunization among the general public and to ensure that parents follow the National Childhood Immunization Programme schedule need to be continued and strengthened.
